# Attitudes, awareness, and perceptions of general public and pharmacists toward the extended community pharmacy services and drive-thru pharmacy services: a systematic review

**DOI:** 10.1186/s40545-023-00525-4

**Published:** 2023-03-02

**Authors:** Bayan F. Ababneh, Siew Chin Ong, Fatema Mahmoud, Louai Alsaloumi, Rabia Hussain

**Affiliations:** 1 Discipline of Social and Administrative Pharmacy, School of Pharmaceutical Sciences, Universiti Sains Malaysia, Penang, Malaysia; 2 Discipline of Pharmacy and Biomedical Sciences, University of Strathclyde, Glasgow, Scotland UK; 3 Discipline of Clinical Pharmacy, Faculty of Pharmacy, Near East University, Nicosia, Northern Cyprus Turkey

**Keywords:** Attitudes, Awareness, Perceptions, Drive-thru pharmacy, Pharmacists, General public

## Abstract

**Background:**

Several extended and newly added pharmacy services were evaluated in different countries. This review aims to provide a summary of studies on attitudes, awareness, or perceptions toward various extended and drive-thru pharmacy services at community settings among pharmacists and the general public.

**Methods:**

To find qualitative and descriptive quantitative studies, that reported on the attitudes, awareness, or perceptions of the general public and pharmacists toward the practice of any extended community pharmacy service and drive-thru pharmacy services in a community setting and conducted from March 2012 to March 2022. Researchers used databases such as Embase, Medline PubMed, Scopus, Web of Science, and Science Direct. The reviewers extracted data independently using the PRISMA checklist.

**Results:**

There were 55 studies found according to the inclusion criteria. Various extended pharmacy services (EPS) and drive-thru pharmacy services were noted in the community setting. Pharmaceutical care services and healthcare promotion services were the noticeable performed extended services. There were positive perceptions and attitudes toward extended and drive-thru pharmacy services among pharmacists and the public. However, some factors, such as lack of time and shortage of staff, affect the practice of those services.

**Conclusion:**

Understanding the major concerns toward the provision of extended and drive-thru community pharmacy services and improving pharmacists’ skills through more training programs to provide such services efficiently. In the future, more reviews for EPS practice barriers are recommended to faceup all concerns and find standardized guidelines by stakeholders and organizations for efficient EPS practices.

**Supplementary Information:**

The online version contains supplementary material available at 10.1186/s40545-023-00525-4.

## Introduction

Pharmacists are considered accessible healthcare providers without the need for appointments to get consultations or counseling [1]. Pharmaceutical care can be applied in all settings, i.e., community, hospital, clinic, and others [2]. Pharmaceutical care at community pharmacy settings added a wide range of services, ranging from providing concise counseling to detailed counseling and other value-added services which are known to be extended pharmacy services [3].

Community pharmacists are widely considered multidisciplinary healthcare providers, and their roles moved to be more patients’ oriented than focusing on products only [4, 5]. In the United States (U.S.), a community pharmacist is considered as an accessible primary healthcare provider [6]. A community pharmacist provides efficient health services which reduce the burden on primary health institutions, for example, administrating vaccinations during outbreaks such as during the time of the H1N1 pandemic in 2009 [7, 8] resulted in an elevation of the number of the pandemic vaccine doses administered and reduction of the time for getting 80% vaccination coverage [8]. Community pharmacists are vital in providing primary healthcare services in Canada, as evidenced by the value of their pharmaceutical care services in several pharmacy practice projects [9]. Moreover, community pharmacy is considered as an ideal setting to provide healthcare services for the public compared to other primary healthcare settings because it is accessible for both urban and rural areas with relatively long opening hours [10–12].

Extended pharmacy services (EPS) are related to the services that are provided at pharmacies other than traditional services (ex. dispensing the prescribed or non-prescribed medications and giving counseling or instructions about dispensed medications). EPS include identifying medication-related problems by doing comprehensive medication reviews, providing some monitoring for diseases such as measuring blood pressure reading or blood glucose level, and contacting the primary healthcare team [3, 13]. Medication-related problems can be identified too by getting involved in disease management by doing a series of services such as detailed patient education about medications or health-related diseases [3, 13]. In addition to the EPS, there are newly added services at pharmacies such as drive-thru service or drive-through service [14].

The first drive-thru pharmacy service was initiated by Walgreens pharmacy in the United States in the 1990s and nowadays it is spread to many countries around the globe [14], such as the United Kingdom [15], Australia [16], Croatia [17], Taiwan [18], Malaysia [19], Jordan [20], Saudi Arabia [21], and Qatar [22]. Drive-thru services were established either at hospital or community pharmacy settings or both, to reduce waiting time in the pharmacies, to improve the availability and provision of healthcare services for the targeted population, and recently to improve safety during COVID-19 [23].

Quality assessment of pharmacy services can be judged by regulatory agencies, consumers, and drug-service providers [24]. Patients’ perceptions toward healthcare service is an indicator of successful implementation of service that can be evaluated by patients’ perceptions toward that service [25, 26]. To determine the quality level of pharmacy service, it is very important to consider the pharmacists’ perspectives toward provided services [27] and compare consumers’ perceptions and expectations with pharmacists’ perceptions and willingness toward healthcare services [28].

Several implemented extended pharmacy services and drive-thru services at the community setting have been evaluated in different countries by exploring pharmacists’ or public’s perceptions toward those services. This review aims to summarize attitudes, awareness, or perceptions toward various extended including drive-thru pharmacy services at community settings among pharmacists and the public, to figure out the extent of the practice of such services, and the factors that affect its provision, so standardized guidelines for efficient practice can be found by stakeholders.

## Methods

This systematic review followed the Preferred Reporting Items for Systematic Reviews and Meta-Analysis (PRISMA) statement, and it was registered with PROSPERO on April 13, 2022 [Registration number: CRD42022314516].

### Eligibility criteria

Table 1 summarizes the inclusion and exclusion criteria. Observational descriptive quantitative and qualitative studies conducted from March 2012 to March 2022 that reported the attitudes, awareness, or perceptions of the general public and pharmacists toward the practice of any extended community pharmacy service and drive-thru pharmacy services in a community setting and were published in peer-reviewed journals in the English language were included.

**Table 1:** Inclusion criteria of studies in the systematic review

Category	Inclusion criteria
Language	Only English
Publication year	March 2012–March 2022
Publication type	Descriptive cross-sectional and qualitative studies
Population	Pharmacists, and the general public or consumers who represent the general public
Phenomenon of interest	Extended services, and drive-thru service in community pharmacy settings all around the world
Outcome measures restricted to	Describe the practice of providing extended services, or drive-thru service in terms of awareness, perceptions, and attitudes
Pharmacy setting	Only community pharmacy setting

A simple definition of attitudes is a mindset or tendency of acting toward something in a certain way according to personal experiences [29]. However, the definition of perceptions is the process by which individuals organize and interpret their impressions toward any kind of information in the environment [29]. Moreover, the definition of awareness is the knowledge that something exists [30].

Exclusion criteria were based on studies that reported attitudes, awareness, or perceptions related to any extended pharmacy service in a hospital context, in grocery shops, or in only certain chain pharmacy or outpatient pharmacy or pharmacy that provided certain EPS promoted by drug companies or any pharmacy provided certain workshops or training regarding EPS and assessed the awareness or knowledge after training. In addition, studies not published in English, reports, commentaries, preliminary studies, pilot studies, editorials, book chapters, systematic reviews, conference abstracts, or meta-analysis were not considered.

### Search strategy

An extensive literature search was conducted to find studies on the attitudes, awareness, or perceptions toward the practice of any extended community pharmacy service and drive-thru pharmacy service among the public or community pharmacists in the following databases: Google Scholar, Embase, Medline PubMed, Scopus, Web of Science, and Science Direct, from March 2012 to March 2022 using keywords and Medical Subject Headings (MeSH) terms. The eligible studies were identified by using the following search keywords: extended community pharmacy services, drive-thru pharmacy, customers, public, consumers, pharmacists, attitude, awareness, and perceptions. Populations were not confined to a single country or location. The search keywords were found in either the title or abstract. These keywords were used in connection with one another using Boolean operators ("OR", "AND") and truncation. A supplemental search was done by hand-searching of bibliography lists from all included papers and receiving email alerts for any newly published relevant papers from pre-specified databases to find other papers that were not identified through the electronic search.

### Study selection

The primary reviewer (BFA) imported the search results from all databases into Mendeley reference software, where duplicates were removed. Study selection was done independently by two reviewers (BFA and LA). Any disagreement or uncertainty regarding the inclusion or the exclusion of the studies during the screening process was resolved by discussion between the reviewers, or consultation with a third reviewer (FM). The first phase was the title and abstract screening conducted by two reviewers (BFA and FM) then the selected studies were verified by the primary reviewer (BFA). In the second phase, two reviewers did full-text screening independently (BFA and LA). The final list of studies was developed as a consensus of all reviewers to determine the reasons for exclusion.

### Data extraction

Two reviewers (BFA and FM) completed data extraction independently using a standardized Excel spreadsheet (Microsoft Corp, Redmond, Washington). Any disagreement was resolved by discussion between the two reviewers, or by consultation with a third reviewer (LA). Data extraction for descriptive cross-sectional studies included the name of the first author, year of publication, study country, study design, study objectives, name of the extended community pharmacy service, study participants, inclusion and exclusion criteria, sample size, method of data collection, data analysis, and a summary of major conclusions. Data extraction for qualitative studies or mixed-method studies included the name of the first author, year of publication, study country, study design, study objectives, name of the extended community pharmacy service, study participants, inclusion and exclusion criteria, sample size, method of data collection, data analysis, major themes, and a summary of major conclusions.

### Quality assessment

Two reviewers (BFA and FM) independently assessed the quality of the included studies (*n* = 55), during the data extraction phase. It was assessed based on the type of the studies, for descriptive cross-sectional studies, the National Institute of Health (NIH) quality assessment tool for observational cohort and cross-sectional studies was used, which includes 9 statements with the following options: yes, no, cannot determine, not applicable, and not reported [31]. Joanna Briggs Institute (JBI) critical appraisal checklist for qualitative studies was used for qualitative studies, including 10 statements with yes, no, unclear, and not applicable options [32]. The Mixed Methods Appraisal Tool (MMAT) was used for mixed methods studies, which includes 5 statements with yes, no, and cannot tell [33].

### Data synthesis

A Synthesis Without Meta-Analysis (SWiM) was conducted on the included studies, due to the heterogeneity of the studies [34]. Studies were grouped based on the assessed extended pharmacy service and based on the targeted population. Extended community pharmacy services among pharmacists were categorized into 5 categories: (1) pharmaceutical care, (2) professional and public health activities, (3) extended pharmacy services for special groups of population, (4) health promotion activities, (5) drive-thru pharmacy services. The studies that assessed pharmaceutical care were grouped under pharmaceutical care category. The studies that assessed any of professional or public health activities such as medication reconciliation service, professional interactions between community pharmacists and general practitioners, public health activities such as providing education about the cessation of smoking, good oral hygiene, and measurements of blood pressure and blood glucose level, and inter/intra-professional activities were grouped under professional and public health activities category. The studies that discussed certain extended pharmacy service for special group of population, such as community Pharmacy Anticoagulation Management Service, pharmaceutical care for patients with dementia, pharmaceutical services for the elderly, pharmaceutical care for osteoporosis, and others were grouped under extended pharmacy services for special group of population category. Studies that assessed health promotion activities such as health-promotion and health education activities, counselling of diabetes, asthma, oral contraceptives, smoking cessation, nutrition and physical activity, oral health, and vaccinations were grouped under the health promotion activities category. Studies that assessed drive-thru community pharmacy service were categorized under drive-thru pharmacy services. Extended community pharmacy services among public or consumers were categorized into 2 categories: (1) extended pharmacy services, (2) drive-thru pharmacy services.

## Results

### Study characteristics

**Fig. 1:**
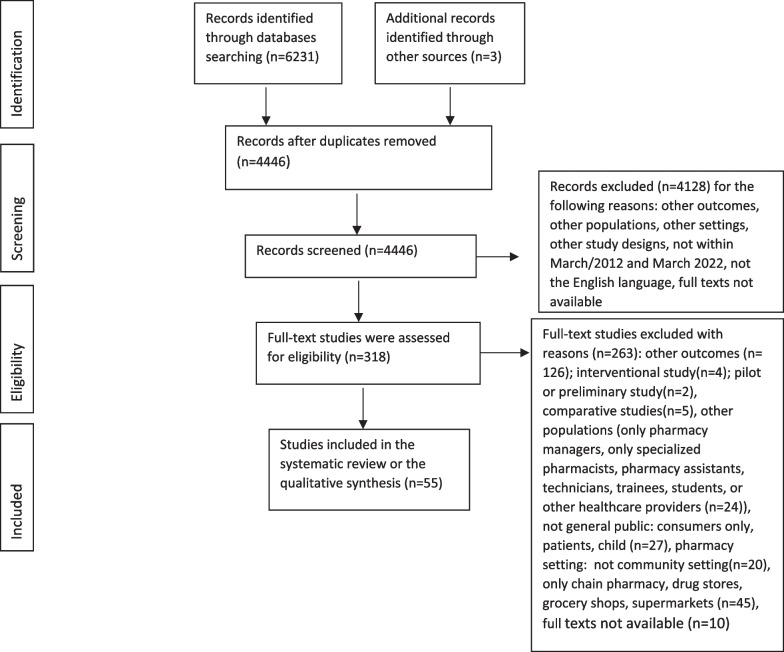
PRISMA flow diagram of the selection process for the systematic review studies

After removing duplicates, a final search strategy from all databases resulted in 4446 total studies. For the full-text screening phase, 318 studies were included. After the full-text screening, 55 studies (49 cross-sectional, 3 qualitative, and 3 mixed-method studies) were accepted for extraction based on the inclusion and exclusion criteria for this review (Fig. 1). A total of 4391 studies were excluded from the study selection process. The extracted data are summarized in Additional file 1.

Of the total 55 included studies, there were 49 descriptive cross-sectional studies, 3 qualitative studies, and 3 mixed-method studies. The included studies were conducted in 26 different countries where five were conducted in Europe (Ireland, United Kingdom (UK), Bulgaria), three in North America (Jamaica, Canada), three in Oceania (Australia, New Zealand), 39 in Asia (Malaysia, Jordan, Yemen, Palestine, Qatar, United Arab Emirates (UAE), Lebanon, China, Iran, Iraq, Egypt, Turkey, Nepal, and Pakistan), and five in Africa (Nigeria, Ghana, South Africa). Malaysia was the most prominent for cross-sectional studies (*n* = 13) and qualitative studies were conducted in Pakistan, the United Kingdom, and Canada. While the mixed-method studies were conducted in New Zealand (*n* = 2) and Australia. Most studies investigated pharmacists’ perceptions (*n* = 52), with only three studies investigated general population perceptions (*n* = 3). Most of the cross-sectional studies used self-administered questionnaires for data collection, while qualitative studies used the semi-structured interviews (*n* = 2) and one used focused group interviews. For the mixed methods studies surveys and interviews have been used.

The included studies are presented in Additional file 1, which shows pharmacists’ awareness, perceptions, or attitudes toward various extended pharmacy services and drive-thru service at community setting including: extended role services [35], pharmaceutical care services [36–44], over-the-counter pharmaceutical services [45, 46], health promotion and health education [47], public health activities [48, 49], value-added services [50], minor aliment services [51], medication reconciliation [52], professional services [53, 54], services for people with mental disorders [55–57], lifestyle assistance services for people with cardiovascular diseases [58], anticoagulation management services [59], services for geriatrics [60], osteoporosis assessment screening [61], back pain management services [62], Human Papillomavirus (HPV) Vaccination Services [63], emergency contraception services [64], clinical services [65], intermediate care services [66, 67], interactions with general practitioners [68], oral health services [69, 70], dermatologic care services [71, 72], travel medicine services [73], smoking cessation counselling [74, 75], weight management services [76], nutrition counselling [77], cancer health promotion [78], breast cancer health promotion [79–84], bowel and breast cancer screening [85], and drive-thru pharmacy services [20]. Studies that showed public’s awareness or perceptions or attitudes toward various extended pharmacy services and drive-thru services at community settings were; weight management services [86], current pharmacists’ role [87], and drive-thru services [88].

Of the 49 included descriptive cross-sectional studies, 19 studies reported the attitudes, 13 studies reported the attitudes and perceptions, 8 studies reported the perceptions, 4 studies reported the attitudes, awareness, and perceptions, 3 studies reported the awareness and perceptions, and 2 studies reported the awareness toward EPS. Of the 3 included qualitative studies, 2 studies reported the awareness and attitudes, and one study reported the attitudes and perceptions toward EPS. Of the 3 included mixed-method studies, 2 studies reported the perceptions, and one study reported the attitudes and perceptions toward EPS.

Table 2 shows details of country which indicated extended services and drive-thru service at community pharmacy setting according to the inclusion criteria of this review. The most noticed service was the pharmaceutical care services (*n* = 9), followed by breast cancer health promotion services (*n* = 6). Pharmacists in Malaysia had represented various extended services at community setting (*n* = 13), followed by Jordan (*n* = 5). Drive-thru service at a community pharmacy setting was performed in Jordan (*n* = 2).

**Table 2:** Summary of extended services and drive-thru service at community pharmacy setting according to the inclusion criteria of this review

Name of pharmacy service	Number of studies	Country published (Reference No.)
Pharmaceutical care service	9	Nigeria [36, 40], China [37], Poland [38], Iran [39], Malaysia [44], Jamaica [41], Jordan [42], Pakistan [43]
Intermediate care service	2	UK (Ireland) [66, 67]
Public health activities	2	Yemen [48], Nigeria [49]
Cancer health promotion	1	Ghana [78]
Breast cancer health promotion	6	Qatar [79], Iraq [84], Jordan [80], UAE [81], Palestine [82], Malaysia [83]
Breast and bowel cancer screening	1	Australia [85]
Drive-thru service	2	Jordan [22, 88]
Travel medicine services	1	Malaysia [73]
Weight management services	2	UK [86], Malaysia [76]
Nutrition counselling	1	Egypt [77]
Mental health services	3	UK [55], Malaysia [56], Canada [57]
Pain management	1	Lebanon [62]
Smoking cessation	2	Malaysia [74], Jordan [75]
professional practice	2	Malaysia [53], UAE [54]
Oral health services	2	Malaysia [69], Lebanon [70]
Minor Aliment services	1	Malaysia [51]
Lifestyle assistance to cardiovascular disease	1	Malaysia [58]
Osteoporosis risk assessment	1	Malaysia [61]
Services for geriatrics	1	New Zealand [60]
Supplementary OTC prescribing	2	China [45, 46]
Clinical services	1	Turkey [65]
Medication reconciliation	1	Jordan [52]
Dermatologic management services	2	Palestine [72], Durban (South Africa) [71]
Health promotion and health education	1	Malaysia [47]
Value-added services	1	Bulgaria [50]
Emergency contraception services	1	Nepal [64]
Vaccination HPV	1	USA [63]
Interactions with General practitioners	1	Malaysia [68]
Extended role	2	Pakistan [35], Jordan [88]
Anticoagulation management services	1	New Zealand [59]

### Methodological quality

Generally, most of the studies got good quality using the assessment tools according to the study design. For descriptive cross-sectional studies, the majority of studies achieved yes for the 9 statements of the National Institute of Health (NIH) quality assessment tool for observational cohort and cross-sectional studies. For qualitative studies, all three studies achieved yes for the 10 statements of the Joanna Briggs Institute (JBI) critical appraisal checklist for qualitative studies. Similarly, for mixed-method studies, all three studies achieved yes for the 5 statements of the Mixed Methods Appraisal Tool (MMAT) (see Additional file 2). These assessment tools used only the yes, no scoring method. For ease of doing the quality assessment for the studies, the reviewers converted yes, no, not reported, not available, and cannot determine scores into numerical scores. We marked 1 for each yes, and 0 for each no or not reported or cannot determine or not available. For descriptive cross-sectional studies, the grading was decided based on the total score out of 9 for each study: 0–3 (poor), 4–6 (fair), and 7–9 (good). For qualitative studies, the grading was decided based on the total score out of 10 for each study: 0–3 (poor), 4–6 (fair), and 7–10 (good). For mixed-method studies, the grading was decided based on the total score out of 5 for each study: 0–1 (poor), 2–3 (fair), and 4–5 (good). Among descriptive cross-sectional studies, 48 studies out of 49 achieved good quality scoring and only one achieved fair quality scoring. Qualitative and mixed-method studies all achieved good quality scoring (see Additional file 3). No studies were excluded based on the methodological quality assessment, as only poor quality studies were planned to be excluded from data synthesis. This approach of excluding poor quality studies from data synthesis was reported in previous systematic reviews [89–91]. Two researchers scored each on the included studies independently. Any disagreements were resolved by discussion.

### Attitudes, awareness, and perceptions of community pharmacists toward extended community pharmacy services

#### Pharmaceutical care

In Nigeria (2014), 95% of community pharmacists perceived that pharmaceutical care is a valuable service that may improve patients’ health and raise consumers’ confidence in the pharmacy profession which will enhance pharmacy practice [40]. In 2019, positive attitudes toward pharmaceutical care (51.2%) were showed among Nigerian community pharmacists’, however, their practice was poor because of a shortage of staff, poor collaboration with other healthcare providers; lack of pharmaceutical care skills, and the time-consuming of pharmaceutical care provision [36]. Compared to Nigeria, in Pakistan (2017), there was poor awareness with positive attitudes toward extended pharmacy services and pharmaceutical care among community pharmacists in Lahore due to a shortage of manpower, training programs, and others [35], and in 2020, they started providing pharmaceutical care (38.9%) to improve consumers’ health outcomes [43]. In Malaysia (2021), community pharmacists in Kuala Lumpur had positive attitudes toward the practice of pharmaceutical care (93.7%) [44]. In Jamaica (2021), community pharmacists showed positive attitudes toward pharmaceutical care as well (> 80%) and the shortage of resources is the main cause of not practicing pharmaceutical care [41]. In Poland (2021), community pharmacists perceived that pharmaceutical care practice is very important but lack of time is the main reason for not providing it all the times [38]. Same practice of pharmaceutical care was documented in China (2016) by community pharmacists [37]. In Iran (2015), pharmacists’ perceived that the provision of pharmaceutical care has large benefits to patients’ treatment outcomes [39]. In Jordan (2012), community pharmacists showed positive attitudes toward pharmaceutical care practice by documenting adequate practice [42].

#### Professional and public health activities

In Jordan (2019), there was poor awareness and practice toward medication reconciliation service in community pharmacies by pharmacists [52]. In Malaysia (2021), interactions between community pharmacists and general practitioners were suboptimal, and more training programs are needed to improve such services [68]. In Turkey (2012), community pharmacists perceived themselves as serving in a clinical consultancy role and advising on drug therapy [65].

Nigerian community pharmacists reported less inclined toward the practice of public health activities due to lack of time, non-cooperative consumers, and shortage of manpower [49]. Compared to Nigeria better results were documented toward public health activities in Yemen as positive attitudes were noticed among community pharmacists, such as providing education about the cessation of smoking and alcohol drinking, good oral hygiene, and measurements of blood pressure and blood glucose level when needed for consumers [48]. In Malaysia (2017), community pharmacists showed positive awareness toward inter/intra-professional activities and pharmaceutical care practice [53]. In UAE (2015), almost all of community pharmacists performed professional practice by contacting physicians to minimize any medications errors and enhance consumers health-related outcomes [54].

#### Extended pharmacy services for special groups of population

In Malaysia (2020), a practice of lifestyle assistance services was documented to the cardiovascular disease patients among pharmacists in the community setting [58]. In New Zealand (2020), community pharmacists showed positive perceptions toward the provision of Community Pharmacy Anticoagulation Management Service (CPAMS), as it may improve pharmacists–patients relationships, and patients health outcomes [59].

Community pharmacists in the UK (2013) perceived positive attitudes and practice toward pharmaceutical care for patients with dementia. The frequently provided services for patients with dementia were initiation and stopping medications, adherence to medication, and checking the availability of formulation types [55]. In Malaysia (2019), there was positive attitudes toward mental healthcare among community pharmacists, but most mentally ill patients were referred mostly to hospitals for healthcare [56]. Additionally, in Canada (2015), pharmacists showed a positive attitude toward services for patients receiving Anti-Depressant Therapy (ADT), especially for medication initiation [57].

A good range of services for the elderly were perceived to be provided by community pharmacists in New Zealand, such as encouraging medications’ adherence by offering compliance packaging, medication review, and repeat prescription reminders, in addition to some screening services such as blood pressure measurements [60].

In Malaysia (2021), there were low osteoporosis risk assessment tools practice of and modest awareness among community pharmacists [61], while in Lebanon (2019) the perceived back pain management services by community pharmacists were dispensing of non-steroidal anti-inflammatory medications and patients’ referral to the physician when needed [62].

#### Health promotion activities

Value-added pharmacy services (VAPS) were offered by Bulgarian community pharmacists and mostly common offered service was blood pressure and glucose level measurement, and the community pharmacists perceived positive attitudes toward VAPS [50]. In Malaysia (2019), community pharmacists showed positive perceptions and attitudes toward pharmacist-led minor ailment services [51]. Additionally, in Malaysia (2014), involvement of community pharmacists in health-promotion and health education activities were high, especially in counselling of diabetes, asthma, oral contraceptives, smoking cessation, nutrition and physical activity, and oral health that could enhance public’s health outcomes [47].

The community pharmacists in Nepal (2020), had positive attitudes and practices toward emergency contraception pills [64].

In the USA (2017), positive practice and attitudes were observed among community pharmacists toward vaccinations including influenza vaccine, herpes zoster, pneumococcal polysaccharide (PPSV23), tetanus/diphtheria/pertussis (Tdap), and Human Papillomavirus Vaccination (HPV) [63].

Malaysian community pharmacists showed positive attitudes toward smoking cessation practice, but more continuing education sessions were needed to improve this practice [74]. Compared to Malaysia same results were documented in Jordan (2022), as community pharmacists showed good attitudes toward smoking cessation practice with low practice due to lack of educational materials, minimal demand by consumers, and low training education programs [75].

In Lebanon (2019), positive attitudes were perceived toward oral healthcare services among community pharmacists, but limited interaction with dentists and insufficient training were major concerns of practicing [70]. In Malaysia (2020), frequent oral health consultations were provided by community pharmacists and showed positive attitudes toward provision of oral health services. The most frequent oral healthcare services they provided were over-the-counter (OTC) treatments and referral to dentists [69].

Egyptian community pharmacists perceived positive attitudes toward their role in nutritional assessment and medical nutrition therapy and stated that they routinely provide this service for patients with the following medical conditions; obesity, diabetes and hypertension [77]. In Malaysia (2019), positive attitudes toward weight management services (WMS) observed among community pharmacists as they regularly provided advices to the consumers about physical activity and healthy eating [76].

In Qatar (2013), pharmacists showed positive attitudes toward breast cancer health promotion practice with low level of practice due to lack of educational materials, and public awareness [79]. In Iraq (2017), community pharmacists showed favorable attitudes toward breast cancer health promotion practice but lack of time is the major factor that reduce the level of practicing this service [84]. Similarly, in Jordan (2016), community pharmacists had positive attitudes toward provision of breast cancer health promotion but insufficiency of time and suitable educational skills lower their involvement in this service [80]. In UAE (2013), despite the low participation in breast cancer health promotion practice among community pharmacists, they perceived that providing such services was helpful for females consumers [81]. Additionally, community pharmacists in Palestine (2021), perceived positive attitudes toward breast cancer health promotion, but their concerns toward practice of this service were shortage of staff, insufficiency of time, and fear of offending the patients [82]. Furthermore, in Malaysia (2012) insufficiency of time and lack proper breast cancer educational materials and training were the major causes of not providing breast cancer health promotion by the community pharmacists [83].

#### Attitudes, awareness, and perceptions of consumers or public toward extended community pharmacy services

In the United Kingdom (2012), the Scottish public showed poor awareness toward healthcare services provided in the community pharmacy setting (13.2%; *n* = 162). They accepted the idea of consulting community pharmacists regarding weight management services, however the lack of privacy at the community pharmacy setting (47.3%; *n* = 592) and the perceived lack of pharmacists’ specialist knowledge were the major concerns toward using this service [86]. In Jordan (2018), public showed poor awareness toward the knowledge of pharmaceutical care services at community pharmacy setting (55.1%), they assured the importance of the role of pharmacists in providing healthcare services other than counselling and dispensing and expect much more services to be provided by community pharmacists at their country [87].

#### Drive-thru community pharmacy services and pharmacists

In Jordan (2017), pharmacists were aware about the concept of drive-thru pharmacy service (n = 194, 85.5%), and the most stated advantage of drive-thru pharmacy service was serving sick customers. Only 27.9% (n = 63) were willing to register with this service. Most of pharmacists believed that drive-thru pharmacy service might affect the image of the pharmacy profession negatively, and make them feel more like a fast food worker than a pharmacist [20].

#### Drive-thru community pharmacy services and consumers or public

In Jordan (2019), there was a good awareness about drive-thru pharmacy service among the public 26.8% (n = 212). The usage of the service was minimal 10.9% (n = 86), and the most recognized concern among the public while using this service was the poor communication that may happen between the pharmacist and the patient. About 59.1% of the public expressed high support for the introducing this service to the community pharmacy practice [88].

Details of the results are presented in additional files [see Additional file 1].

## Discussion

The present systematic review aimed to provide a summary of studies on attitudes, awareness, or perceptions toward various extended and drive-thru pharmacy services at community settings among pharmacists and the public.

In this review, it is noted that there is a positive shift toward patients’ health outcomes services at community pharmacy setting. This shift is very helpful for patient healthcare outcomes and may improve the interactions between all healthcare providers [92]. Contacting physicians by community pharmacists to minimize medications errors and improve health-related outcomes was noted in UAE [54]. The awareness toward interaction between community pharmacists and general practitioners (GPs) was noted in Malaysia, but the practice of such service was suboptimal [68]. To solve this malpractice, teaching programs at universities for undergraduates will improve the interactions between pharmacists and GPs [93].

Positive attitudes were noted among community pharmacists toward public health activities such as providing education about some health conditions, measurements of blood pressure and blood glucose level at community pharmacy [48]. Additionally, a good practice was noted in Malaysia among community pharmacists toward health education activities for some health conditions such as diabetes education, asthma education, oral health education, and others that could play an important role in enhancing public’s health outcomes [47]. However, there were poor attitudes toward public health activities among community pharmacists in Nigeria due to some factors such as lack of time, non-cooperative consumers, and shortage of manpower [49]. These factors could be improved through enhanced awareness of public toward such service, and increasing community pharmacists’ incentives [49, 94].

Studies in this review, also have shown positive attitudes toward pharmaceutical care among community pharmacists in Nigeria, Malaysia, Jamaica, Poland, China, Iran, Jordan, and Pakistan [35–38, 40–42, 44, 95]. However, it is noted that some factors prevent community pharmacists provide pharmaceutical care all the time. For example, it is observed that lack of time or staff or resources shortages are the main barriers to its practice in Nigeria, Jamaica, Poland, and Pakistan [35, 36, 38, 41]. Professional skills to shift pharmacists’ traditional roles into extended roles are essential [96].

In this review, positive attitudes and perceptions were noted toward various EPS that are related to a special group of populations such as services for geriatrics, services for mental disorders, cardiovascular risk assessment, osteoporosis risk assessment, dermatologic care services, cancer health promotion, and infectious care services such as vaccinations, and travel medicine service [55–57, 60, 61, 63, 71, 73, 79–81, 84, 85, 97]. These specialized extended pharmaceutical services can be promoted by various specialized training courses of the Board of Pharmacy Specialties (BPS), such as geriatrics, psychiatric, cardiology, pharmacotherapy, oncology, and infectious [98].

It was noted that there is a lack of educational courses, and training programs for pharmacists, to practice smoking cessation services confidently [74, 75]. Healthcare providers who have received training programs for smoking cessation services will practice such services more than those who did not [99]. Other training programs that listed under continuous pharmacy education, such as anticoagulation program, prepare pharmacists to practice these extended pharmacy services smoothly and enhance patients’ health outcomes [100]. Comparable findings were noted in New Zealand; by application of The Community Pharmacy Anticoagulation Management Service (CPAMS), better anticoagulation control were noticed and community pharmacists who were involved in CAMPS showed positive attitudes toward it [59, 101].

Furthermore, it is noted that public had positive attitudes and perceptions toward EPS which are accessible and helpful for them. However, poor awareness toward EPS and some concerns toward its provision by community pharmacists where highlighted by public such as the lack of pharmacists’ specialized skills for providing EPS [86, 87]. Therefore, it is important to improve pharmacists’ skills to provide such extended services through training programs. Findings toward perceptions of various EPS practices among pharmacists and the public were positive, but more training and experienced skills are needed to provide such services and promote pharmacy practice. Comparable findings were noted in a previous systematic review of EPS as positive perspectives were observed with noted barriers that inhibit such performance, and more training programs are needed to improve EPS practice [102].

A good awareness was noted toward drive-thru service at community setting among pharmacists and public [20, 88]. However, some concerns were noted while using this service, such as the poor communication between the pharmacist and the patient; this can be intervened by applying telemedicine [103], and developing of standard drive-thru model to facilitate its application efficiently [23].

## Limitations

This study is not devoid of limitations. We limited our review retrieval to Embase, Medline PubMed, Scopus, Web of Science, and Science Direct search engines, English language published studies, and studies that published between March/2012 and March/2022. Although we have done cross-referencing, which added a few studies in our review, we still cannot claim that we had access to all the relevant articles related to this topic. Several limitations were noted in the included studies. For instance, most cross-sectional studies utilized a self-administered questionnaire to assess perceptions and awareness, so results are prone to measurement bias.

## Conclusion

Most included studies showed good awareness or perceptions toward EPS and drive-thru pharmacy service in community settings among pharmacists or the public. These services are beneficial for health-related outcomes. Ultimately, understanding the major concerns toward providing these services and improving pharmacists’ skills through more training programs to provide such services efficiently. In the future, more reviews for EPS practice concerns are recommended, to solve those concerns and find standardized guidelines by stakeholders and organizations for efficient EPS practices.

## Supplementary Information

**Additional file 1. **Characteristics of the included studies in the review.

**Additional file 2. **Quality assessment details of included studies in this systematic review.

**Additional file 3. **Quality assessment grading of included studies in this systematic review.

## Data Availability

All data generated or analyzed during this study are included in this published article (and its additional files).

## Abbreviations

EPS: Extended Pharmacy Services
UK: United Kingdom
UAE: United Arab Emirates
PRISMA: Preferred Reporting Items for Systematic Reviews and Meta-Analysis
MESH: Medical Subject Headings
NIH: National Institute of Health
JBI: Joanna Briggs Institute
MAAT: Mixed Methods Appraisal Tool
SWiM: A Synthesis Without Meta-Analysis
HPV: Human Papillomavirus Vaccination
PPSV23: Pneumococcal polysaccharide
Tdap: Tetanus/diphtheria/pertussis
OTC: Over the counter
GPs: General Practitioners
ADT: Anti-depressant therapy
CPAMS: Community Pharmacy Anticoagulation Management Service
VAPS: Value-Added Pharmacy Services
BPS: Board of Pharmacy Specialties
WMS: Weight Management Services
