# Supplementary material for: Attitudes, awareness, and perceptions of general public and pharmacists toward the extended community pharmacy services and drive-thru pharmacy services: a systematic review
Source: J Pharm Policy Pract. 2023 Mar 2;16:37. doi: 10.1186/s40545-023-00525-4 (PMC9979876; doi:10.1186/s40545-023-00525-4)
Supplement: Supplementary file 1 — Additional file 1. Characteristics of the included studies in the review. [file 40545_2023_525_MOESM1_ESM.pdf]

Additional file 1: Characteristics of the included studies in the review

Author, Year	Country	Study design	Method of data collection	Main objectives	Population	Sample size	Key findings include awareness, attitudes, and perceptions
Anna Millar,2016	Ireland	CS	Questionnaire	To understand CPs' awareness of and involvement in IC	Pharmacists	190	**Awareness:** Three-quarters of pharmacists were either not involved or unsure if they were involved with providing services to intermediate care services (IC)<br>**Perceptions:** A small minority (1.2 %) of CPs reported that they received communication regarding medication changes made in hospital or IC settings 'all of the time'<br>Only 9.5 and 0.5 % of respondents 'strongly agreed' that communication from hospital and IC, respectively, was sufficiently detailed<br>**Attitudes:** (81.6 %) CPs indicated that they would like to have greater involvement with IC services
Seena A. Yousuf,2019	Yemen	CS	Questionnaire	To explore attitudes and practices of CPs in public health activities	CPs	200	**Attitudes:**62.3% of the pharmacists had a positive attitude toward participation in public health activities<br>**Perceptions:** Providing education to stop tobacco chewing, smoking, and alcohol drinking, and improve oral hygiene was an important activity of the CPS<br>Blood pressure measurements (86%, n = 172) and glucose tests (45%, n = 90) were commonly conducted for clients<br>Lack of time (71%, n = 142) and lack of teamwork (70%, n = 140) were mentioned as common barriers to participation in public health activities
Maguy El Hajj,2013	Qatar	CS	Questionnaire	To explore attitudes and practices of CPs in breast cancer health promotion	CPs	190	**Attitudes:** More than 60% were highly interested in being engaged in breast cancer health promotion activities<br>87% believed that discussing breast cancer awareness with female patients in the pharmacy was beneficial to patient<br>**Perceptions:** Eighty-eight percent of CPs indicated that they never invited healthcare professionals to provide breast cancer education<br>78% said that they never distributed breast cancer educational materials, and 58% reported that they never counseled patients about breast cancer<br>Pharmacists perceived many barriers for integrating breast cancer health promotion into their daily practice including lack of educational materials (79%) and lack of public recognition (61%)

Rana Abu Farha,2017	Jordan	CS	Questionnaire	To investigate awareness, perception, and barriers to drive-thru pharmacy services among pharmacists	Pharmacists	250	**Awareness:** The majority of pharmacists reported were aware about the concept of drive-thru pharmacy service (n = 194, 85.5%)<br>**Attitudes:** Only 27.9% (n = 63) reported that they are willing to register with this service<br>**Perceptions:** The most important advantage of drive-thru pharmacy service was serving sick<br>Most of pharmacists agreed that drive-thru pharmacy service may negatively affect the image of pharmacy profession (n = 168, 74.6%), and it makes pharmacists feel more like a fast food workers than a pharmacist (n = 147, 65.9%)
Khawla Abu Hammour,2019	Jordan	CS	Questionnaire	To evaluate public consumers' awareness and perceptions towards the drive-thru pharmacy service	Public	789	**Awareness:** 26.8% (n = 212) of the customers knew that the drive-thru pharmacy service was newly implemented in Jordanian pharmacies<br>**Attitudes:** Only 10.9% (n = 86) reported to have used this service<br>Customers (59.1%) expressed their support for the introduction of this service to pharmacy practice in the country<br>**Perceptions:** Busy customers seem to use this service more (men being married and having children). Hence, this service seems to be most beneficial to a certain type of customers. However, concerns about poor communication between the pharmacist and the patient represented the main disadvantage of the drive-thru service.
Nur Akmar Taha,2016	Malaysia	CS	Questionnaire	To assess CPs' knowledge, attitudes, and practices (KAP) in travel medicine advice	CPs	111	**Awareness:** Most of the respondents (82%) were not trained in travel medicine advice<br>**Attitudes:** The majority had a positive attitude towards travel medicine advice<br>A vast majority provided travel medicine advice mainly to adults who travel as tourists, and the primary travel advice given was on travelers' diarrhoea
Anita Weidmann,2012	UK, Scotland	CS	Questionnaire	To describe public views of weight management services	Public	1236	**Awareness:** Most respondents lacked awareness of the types of health services available through community pharmacy (13.2%; n = 162)<br>**Attitudes:** (25%; n = 320) would not feel comfortable speaking to a pharmacist or medicines counter assistant about weight-related issues<br>**Perceptions:** Around one-third agreed of public (35%; n = 438) that it was more convenient to obtain weight management advice from a pharmacist than it is to make an appointment with a GP<br>Concerns over privacy (47.3%; n = 592) and perceived lack of pharmacists' specialist knowledge
Heather E. Barry,2013	UK	CS	Questionnaire	To explore CPs' experiences and attitudes towards people with dementia	CPs	182	A greater proportion of pharmacists provided pharmaceutical care to people with dementia living at home (91.2%) than those living in care homes (40.1%)<br>Respondents most frequently encountered queries relating to starting and stopping medications, compliance with medication, and availability of formulation types

							**Attitudes:** A positive attitude towards people with dementia, and respondents demonstrated a strong person-centered approach towards this patient population
Nur Taha,2014	Malaysia	CS	Questionnaire	To assess CPs' knowledge, attitudes, practices, and perceived barriers to tobacco cessation counselling	CPs	85	**Attitudes**: Most Pharmacists showed positive attitudes toward tobacco cessation<br>The practice of tobacco cessation counselling was limited mainly to advising patients to quit smoking<br>**Perceptions**: The main barriers to tobacco cessation counselling included a lack of patient demand (71.8%), lack of education materials (69.4%) and lack of time (68.2%)
Abdul Nazer Ali,2017	Malaysia	CS	Questionnaire	To describe pharmacists' attitude towards professional practice	CPs	87	**Attitudes**: Majority of the respondents were having good attitudes towards managerial, dispensing and maintenance of competence activities<br>**Perceptions:** Only minority of the respondents realized the importance of establishing professional relationship with doctors<br>Continuing education and training programs on professional practice would be one significant approach in equipping community pharmacists with the knowledge, skills and behavior needed to expand their scope of practice and improving public healthcare outcomes
Ali Blebil,2020	Malaysia	CS	Questionnaire	To assess knowledge, attitudes, practice, and skills of CPs toward oral health services	CPs	206	Half of the pharmacists provided two to five oral health consultations per week and two to five over-the-counter (OTC) oral health products recommendations per week<br>The main services provided by community pharmacists in were the provision of OTC treatments (93.7%), referral of consumers to dental or medical practitioners when appropriate (82.5%), and identify signs and symptoms of oral health problems in patients (77.2%)<br>**Attitudes**: More than 80% of the pharmacists viewed positively and supported integrating oral health promotion and preventive measures into their practices<br>**Perceptions:** The most commonly reported barriers to extending the roles of pharmacists in oral health care include lack of knowledge or training in this field, lack of training resources, and lack of oral health educational promotion materials
Aline Hajj,2019	Lebanon	CS	Questionnaire	To assess knowledge, attitude, and practice among CPs towards dental care	CPs	497	**Attitudes:** 39% of pharmacists has positive attitude and 47.3% a good practice regarding oral health<br>**Perceptions:** Most of the pharmacists (86.52%) perceived oral health promotion as an important part of their services<br>Moreover, 28.77% (n = 143) of pharmacists declared having difficulties in obtaining oral health information<br>The main barriers to a good practice included limited interaction between dentists and pharmacists and lack of training regarding oral health

Amibor Chiedu,2019	Nigeria	CS	Questionnaire	To determine CPs' attitude and practice of PC	CPs	32	Reasons for not practicing PC ranged from lack of personnel (21.9%), lack of collaboration with other healthcare providers; lack of space and non-acceptance by physicians and nurses were 18.8% each, lack of pharmaceutical care skills (9.4%) and pharmaceutical care is time consuming (6.5%)<br>**Attitudes:** Attitude of pharmacists was positive (51.2%), practice was poor (39.0%)
Amutha Selvaraj,2019	Malaysia	CS	Questionnaire	To evaluate CPs' perceptions, attitudes, and barriers of pharmacist-led minor ailment services	CPs	305	**Attitudes:** The majority of CP in Malaysia had positive attitudes towards pharmacist-led minor ailment services<br>**Perceptions:** The majority of CP in Malaysia had positive perceptions towards pharmacist-led minor ailment services<br>The most common perceived barriers were lack of patient's medical information, absence of dispensing separation and lack of support from other healthcare professionals
Carolina Oi Lam Ung,2016	China	CS	Questionnaire	To explore CPs' knowledge, attitudes, practice, and perceived barriers regarding PC	CPs	102	**Attitudes:** While 95 % of the participating pharmacists agreed that patients' health was their primary responsibility, only 57 % believed that they can provide better PC in the future<br>The majority spent most of their work time counselling patients (90 %) and checking prescription (70 %).<br>Only a small portion monitored adverse drug reaction and drug compliance (44 %), engaged in health screening or drug safety promotion (20 %)<br>Insufficient communication with physicians (90 %), lack of time (79 %) and lack of physical space at the pharmacy (76 %) were considered the most significant barriers
Dorota Kopciuch,2021	Poland	CS	Questionnaire	To evaluate the pharmacists' attitudes towards practice in, and knowledge of PC	CPs	400	**Attitudes:** 72% believed PC provision was necessary to ensure pharmacotherapy<br>safety<br>Only 63% of the pharmacists believed that preventing and solving health-related and drug therapy problems for patients were their responsibilities<br>The main reason for non-provision of PC by the pharmacists was the lack of time for such activities, lack of legal regulations, and lack of organizational facilities<br>69% stated that their patients frequently ask them for advice on pharmacotherapy, and 32% contact a physician if they suspect a drug interaction
Gholamhossein Mehralian,2015	Iran	CS	Questionnaire	To evaluate pharmacists' practice and attitude toward PC	CPs	986	The frequency of the appropriate provision of PC was less than 50% for making contact with physician and medication monitoring<br>**Attitudes:** Speaking about attitude, the complete agreement was expressed only by 30.6%<br>The main barrier to PC was highlighted to patients' reluctance in receiving PC
Hasan H. AL-Behadily,2017	Iraq	CS	Questionnaire	To assess CPs' knowledge, attitude, and perceived barriers towards health promotion of breast cancer	CPs	300	**Attitudes:** Mean score for pharmacist attitude was 26.44± 3.86 points, categorizing the overall attitude as favorable<br>**Perceptions:** Lack of time was perceived by a great proportion of pharmacists (68.2%) as a major barrier to providing patient education

Hee Peng Sia,2020	Malaysia	CS	Questionnaire	To assess CPs' perception and practice of lifestyle assistance to patients with cardiovascular disease	CPs	182	Most of the cardiovascular disease services (CVD) investigated were available in the pharmacy except for advice on alcohol consumption (52.7%) and screening and/or monitoring services for obesity (57.7%), which are less likely to be available<br>**Attitudes**: For most of the listed activities in relation to the provision of lifestyle assistance to patients with CVD, the majority of the pharmacists responded that they did it "sometimes" or "most of the time<br>**Perceptions:** Over 75% of the pharmacists responded with "agree to strongly agree" for most of the statements on perception towards the provision of lifestyle assistance to CVD patients<br>>80% of the pharmacists identified lack of time/heavy workload and lack of staff as the main potential barriers in the provision of lifestyle assistance
Ibrahim Rayes,2015	United Arab Emirates	CS	Questionnaire	To explore perception of CPs' toward professional role	CPs	198	The highest prescribed items by pharmacists are vitamins(89.9%, n = 178) followed by cough remedies (84.8%,n = 168) and NSAIDs (72.7%, n = 144)<br>Almost all the participants directly approached physicians in cases of any clarifications about medicines listed in prescriptions or any possible drug-drug interaction (91.9%n = 182)<br>Almost half of the respondents (49.5%, n = 98) spend an average of 3–5 min with patients for explanation and consultation<br>**Perceptions:** Interestingly, about half of the respondents (48.5%,n = 96) consider themselves chemists and 35.4% (n = 70) perceive themselves pharmacologists<br>They consult patients on the use of nutritional supplements, 58.6% (n = 116) consult patients on different choices of diet plans, and 49.5% (n = 98) answer questions about skin care.
Jezreel Francis,2021	Malaysia	CS	Questionnaire	To investigate CPs' awareness &practice of osteoporosis risk assessment tools	CPs	284	**Awareness:** Only a small proportion of pharmacists (14.9%) regularly used osteoporosis assessment tools in their practice<br>**Perceptions:** They perceived these tools to be relevant and beneficial, but perception towards their accessibility, ease-of-use, and administration time was mixed, suggesting unfamiliarity
Kofi B Mensah,2020	Ghana	CS	Questionnaire	To examine CPs' perception and perceived barriers of cancer health promotion services	CPs	436	**Attitudes:** The majority of CP (77.30%) believe that cancer health promotion is an important part of their daily practice<br>**Perceptions:** Pharmacists' perception towards cancer health promotion was generally positive<br>CPs' perception was favorable. Majority (77.3%) perceived integrating cancer health promotion into their daily practice is important for them as CP<br>Lack of cancer educational materials (69%) was the major perceived barrier in providing cancer health promotion services

M Zakour Khadari,2021	Malaysia	CS	Questionnaire	To evaluate CPs' attitudes towards professional practice	CPs	223	**Attitudes:** The highest of CP got a good attitude was 93.7% for PC activities<br>The highest attitudes PC services were attempted to identify any drug-related problem that patients may be experiencing (64.1%) and refer the patient to the doctor when necessary (63.7%)
M. Medhat,2020	Egypt	CS	Questionnaire	To investigate CPs' role in nutrition counseling	CPs	368	**Perceptions:** A total of 255 (69.3%) of CPs considered medical nutrition therapy a part of a pharmacist's duties<br>Only 146 (39.7%) believed that dietary supplements positively impact public health.<br>According to 321 (87.2%) of the participants, the lack of pharmacists' expertise in nutrition counseling was the main barrier to nutrition counseling
MA'AJI Usman,2014	Nigeria	CS	Questionnaire	To evaluate the knowledge, attitude, and practice of CPs towards PC services	CPs	40	**Attitudes:** There is a positive attitude towards the practice of PC<br>97.5% of respondents had knowledge deficit of PC concepts
Mariam K Dabbous,2019	Lebanon	CS	Questionnaire	To determine CPs' knowledge, attitude, and of low back pain treatment	CPs	320	**Perceptions**: Perceived practice toward back pain management among pharmacists was oral therapy was the most prescribed dosage form for back pain compared to local patch and creams. Among oral dosage forms, non-steroidal anti-inflammatory drugs were the most prescribed medications (42%).<br>**Attitudes**: Other perceived attitude was patients' referrals to the physician, if necessary, 73.1 % of the referrals were done by pharmacists
Menghuan Song,2015	China	CS	Questionnaire	To investigate CPs' attitude, practice, and perceived barriers of OTC	CPs	280	**Attitudes:** Overall, a positive attitude towards provision of PC was demonstrated among pharmacists<br>56.8 % believed and 11.8 % completely believed that they would do their best to provide OTC western medicine PC<br>Lack of "scientific evidence of over-the-counter western medicine'' (42.9), and ''Lack of time'' (40.0 %) as the main barriers
Menghuan Song,2017	China	CS	Questionnaire	To investigate CPs' perceptions of OTC traditional Chinese medicine (TCM) pharmaceutical services	CPs	280	**Attitudes:** Pharmacists generally showed positive attitude towards OTC TCM pharmaceutical services<br>They indicated that they acted proactively to find out all the medicines taken by their patients and to remind consumers of possible OTC TCM adverse reactions<br>**Perceptions:** The three main barriers hindering the provision of OTC TCM pharmaceutical service identified in this study were "insufficient professional knowledge" (54.6%), "ambiguity of the professional role of pharmacists" (54.6%) and "lack of scientific evidence of OTC TCM" (45.4%)<br>The three main actions considered most relevant to improving pharmaceutical service of OTC TCM were "formulating or refining legislation to clarify the legal and professional role of pharmacists with respect to TCM" (60.7%), "strengthening training of pharmacists with respect

							to TCM” (57.9%), and “promoting public awareness of the pharmacist’s role” (53.6%)
Nehad M. Ayoub,2016	Jordan	CS	Questionnaire	To evaluate CPs' knowledge, attitudes, and perceived barriers towards breast cancer health promotion	CPs	1000	**Attitudes:** Pharmacists showed favorable attitude towards involvement in breast cancer education<br>Lack of time was perceived by most pharmacists (63.9%) as a major barrier and other highly recognized barriers were lack of privacy (57.1%) and lack of proper educational skills (56.2%)
Ogochukwu Offu,2015	Nigeria	CS	Questionnaire	To assess CPs' knowledge, attitude, and practice of public health activities	CPs	40	**Attitudes:** Pharmacists scored below satisfactory attitude on the practice of public health activities<br>Reported barriers to the practice of public health include inadequate funds, lack of time, lack of space, cooperation of clients, inadequate staff, government regulation, insufficient knowledge, and remuneration.
Osama Ibrahim,2013	United Arab Emirates	CS	Questionnaire	To assess CPs' attitudes, and practice in breast cancer health promotion activities	CPs	275	**Attitudes:** Ninety-six percent of pharmacists indicated that they never invited healthcare professionals to provide breast cancer education in the pharmacy, 67% said that they never distributed breast cancer educational materials and 47% reported that they never counseled patients about breast cancer<br>More than 75% were highly interested in being engaged in breast cancer health promotion activities, and 87% believed that discussing breast cancer awareness with female patients in the pharmacy was beneficial to patients<br>Perceived barriers for integrating breast cancer health promotion into their daily practice including lack of educational materials (87%) and lack of enough time (74%)
Ozlem Erdogan,2012	Turkey	CS	Questionnaire	To CPs' perceptions and attitudes of clinical issues	CPs	49	**Perceptions**: The top clinical issue pharmacists' consulted for by patients were on medication use (73.3%) followed by therapy choice (36.8%) and which physician they should visit (31.6%)<br>CPs perceive themselves as serving in a clinical consultancy role and advise on drug therapy
Ramzi Shawahna,2021	Palestine	CS	Questionnaire	To explore CPs' knowledge, attitudes, beliefs, and barriers toward breast cancer health promotion	CPs	200	**Attitudes:** CP had positive attitudes toward breast cancer health promotion<br>Lack of reimbursement, lack of enough personnel, lack of time, and fear of offending the patients were the main barriers to breast cancer health promotion

Rana Abu Farha,2019	Jordan	CS	Questionnaire	To evaluate CPs' awareness and perception about medication reconciliation service	CPs	121	**Awareness:** Around 31% of pharmacists have a misconception that the medication reconciliation process should be performed only at the inpatient setting.<br>Only 19.8% (n = 24) of the pharmacists stated that they ask all patients for a complete current medication list of medications when they arrive at the pharmacy site<br>**Perceptions:** Medication histories for most patients were lacking information about the dosage, route, frequency, and time of the last refill for each medication listed<br>"Patients lack of awareness about all the medications they are receiving" was the main barrier discouraging community pharmacists from collecting medication histories and participating in reconciliation service
Rania E. Ghanem,2020	Palestine	CS	Questionnaire	To assess CPs' knowledge, attitudes, and practice toward Acne vulgaris management	CPs	270	**Attitudes:** Pharmacists have positive attitude regarding Acne vulgaris (AV) management, but inadequate knowledge was reflected on their treatment practices<br>Only 10% of participants independently dealt with AV without referral to physicians
Rohit Kumar Verma,2019	Malaysia	CS	Questionnaire	To assess CPs' attitudes, practices, and perceived barriers to weight management services	CPs	563	The CPs reported that they generally often/always provided various services related to anthropometrical and physiological measurements (ranged from 52.4 to 90.8%)<br>They also reported that they often/always provided patients with advice on physical activity (n = 510, 92.8%) and healthy eating (n = 515, 93.7%), as well as selling weight loss products (n = 481, 87.5%) and weight loss drugs (n = 379, 69%) as part of their weight management practices.<br>**Attitudes:** the majority of CPs regarded overeating and sedentary lifestyles to be the main contributory factors to overweight and obesity, and that exercise training to be the most effective intervention
Rose Evans,2021	Jamaica	CS	Questionnaire	To determine CPs' knowledge, attitude, and practice towards PC	CPs	193	**Attitudes:** Most pharmacists demonstrated positive attitude (>80%) towards PC, but <50% of pharmacists provided PC<br>88 (45.6%) CPs always or most of the time screened patients for blood pressure and blood glucose; 43 (22.3%), 84 (43.5%) and 84 (43.6%) for always and most times practicing PC activities related to the creation of patient medical records, reviewing patient data and making medical leaflets respectively<br>The most significant barrier found to prevent the practice of PC was the lack of resources (time, work force, and physical infrastructure)
Salah AbuRuz,2012	Jordan	CS	Questionnaire	To know CPs' attitudes, practice, and barriers towards PC services	CPs	291	**Attitudes:** The level of reported pharmaceutical care activities was limited. In general pharmacists have very good attitudes toward PC<br>Providing advice/ education on the appropriate medicines use or side effects was the most frequently reported PC activity<br>The level of practice of drug therapy review, DRP resolution, care plan development and patient monitoring was adequate<br>The respondents were poor at documenting drug therapy

							monitoring, and they seldom offered their feedback to the physician about the patient's progress and outcome
Samir Sakka,2022	Jordan	CS	Questionnaire	To assess CPs' knowledge, attitudes, and practice toward smoking cessation counselling	CPs	150	Pharmacists' mean smoking cessation practice was 2.01+0.64<br>**Perceptions**: Barriers to providing cessation interventions included the lack of training on nicotine replacement therapy products (86%), the lack of smoking cessation programs (84%), and low demands from smokers (83.3%)
Semira A Beshir,2012	Malaysia	CS	Questionnaire	To examine CPs' knowledge, perception, and barriers of breast cancer health promotion services	CPs	35	None of the pharmacists was currently involved in breast cancer health promotion activities<br>**Perceptions:** Lack of time (80%), lack of breast cancer educational materials (77.1%) and lack of training (62.9%) were the top three mentioned barriers<br>Despite these barriers, 94.3% (33) of the community pharmacists agreed that they should be involved in breast cancer health promotion activities
Semira A. Beshir,2014	Malaysia	CS	Questionnaire	To examine CPs' practice, perception, and barriers in health promotion and health education	CPs	40	CPs were actively involved in diabetes counselling (95%), asthma counselling (93%), oral contraceptives counselling (93%), smoking cessation counselling (83%), nutrition and physical activity counselling (78%) and oral health counselling (63%). However, their involvement in health-promotion activities such as immunization (40%) was limited<br>**Perceptions:** Among the pharmacists who deliver health-promotion services, only 15% maintain logbook of this activities and not more than 53% have access to quality evidence on health promotion and obtain feedback from the community<br>A number of challenges such as lack of time (60%) and training (40%) were cited as obstacles that have hindered pharmacists' involvement. Despite these challenges, the participants believe that their involvement in health-promotion activities could improve the public's health and the state of their profession
Stefan Balkanski,2019	Bulgaria	CS	Questionnaire	To evaluate CPs' practice, and attitudes of value-added pharmacy services	CPs	233	Over 51% of CPs offer value-added pharmacy services (VAPS) but mainly measuring of blood pressure (67.4%) and blood glucose (12.9%)<br>About 70% of them believe that patients would rate VAPS as useful<br>**Attitudes:** The community pharmacists (50.4%) had positive attitudes to offering VAPS in pharmacies where most customers were adults

Sujyoti Shakya,2020	Nepal	CS	Questionnaire	To explore CPs' knowledge, attitude, and practice toward extended pharmacy services	CPs	277	Approximately 75% of pharmacists had a good practice on dispensing emergency contraception (ECPs) and 70% of them counselled all the users<br>**Attitudes:** Majority of the respondents had a positive attitude towards ECP<br>(93.4%)<br>**Perceptions:** More than half of the study respondents believed that ECPs are safe to use (53.4%)
Tareq L. Mukattash,2018	Jordan	CS	Questionnaire	To assess public's views and attitudes towards the current role of pharmacists	Public	1214	**Perceptions:** A relative majority of public considered the most important activity performed by pharmacists to be dispensing medications (46.2%), followed by patient counseling (34.6%)<br>The majority of public (86.4%) believed that pharmacists have a role in providing healthcare services, and 68% of respondents reported that in order to serve their needs, a pharmacist must consider the patient's needs and engage patients in determining medication timing and options
Tessa J. Hastings,2017	USA	CS	Questionnaire	To explore pharmacists' practice, barriers, and attitudes toward Human Papilloma Vaccination services	CPs	154	73.4% of pharmacists reported offering at least one vaccination in the previous 12 months. Of these pharmacies, influenza was the most common vaccine provided (94.7%), followed by herpes zoster (83.2%), pneumococcal polysaccharide (PPSV23)(53.1%), and tetanus/diphtheria/pertussis (Tdap) (42.5%) most (82.6%) did not encounter any patients requesting information about the HPV vaccine Further, 89% of pharmacies had not made any recommendations to male or female patients or parents of patients regarding the need for the HPV vaccine<br>**Attitudes**: A large percentage (47.3%) of pharmacists strongly agreed that vaccination against HPV is the best protection against cervical cancer
Yasmeen Thandar,2019	Durban, South Africa	CS	Questionnaire	To explore CPs' knowledge, attitude, and practices regarding complementary services for Atopic Eczema (AE)	Pharmacists	82	43% of pharmacists recommended complementary medicine (CAM) for atopic eczema (AE)<br>Despite 51% reporting that patients do ask about CAM for AE, 54% are not confident discussing or initiating discussions with patients<br>**Attitudes:** Most were interested in broadening their knowledge on CAM and felt it would better prepare them in counselling their patients
Yin Wong,2019	Malaysia	CS	Questionnaire	To assess CPs' perceptions toward practices, and barriers of mental healthcare services	CPs	96	Thirty-one (32.3%) pharmacists did not stock any psychotropics at all, and 40% of these referred patients with mental disorders to hospitals,<br>**Perceptions:** 80% agreed that mental illness is nothing to be ashamed of, and respondents were overall neutral with regard to their perception of patients with mental disorders, and of mental illness<br>The most frequently cited barrier was the lack of knowledge about mental disorders. Others included patients' lack of understanding of PC and legal requirements

Zelal Kharaba,2020	Pakistan	CS	Questionnaire	To explore CPs' perceptions of PC services	CPs	18	**Awareness:** 38.9% CPs reported that they were sometimes provided PC and made efforts to improve their patient's outcomes<br>**Perceptions:** 94.4 % agreed that patients require counseling by pharmacists, and many of them (83.3 %) were involved in educating patients<br>Only 38.9 % spent enough time on each patient and majority have not been instructing their patients about drug and/or food interaction (88.9 %) and side effects of drugs (83.3 %)<br>Pharmacists have poor participation in maintaining documentation
Alamin Alabid,2021	Malaysia	CS	Questionnaire	To evaluate CPs' awareness toward their roles in interaction with general practitioners	CPs	111	57.5% of CPs mentioned that they rarely or never interacted with general practitioners (GPs)<br>**Awareness:** Many CPs (83.5%) were aware of their role in providing patient education, and (85.8%) indicated that they could suggest nonprescription medicines to patients, whereas (69.3%) were aware that they could suggest alternative medicines<br>(59.8%) were aware of their ability to design and regulate prescribed regimens<br>CPs' had awareness toward their roles in providing healthcare for the 16 activities
Furqan K. Hashmi,2017	Pakistan	Qualitative	Semi-structured interview	To explore CPs' perception and attitude of extended role services	CPs	12	**Awareness:** A lack of awareness of majority of CPs about the EPS and<br>PC<br>**Attitudes:** CPs had a good attitude toward practice change and implementation of EPS<br>Barriers are: Lack of training programs for CP, poor salary structures, insecurities in job, shortage of pharmacist and renting out of pharmacists' practicing licenses
Anna Millar,2016	Northern Ireland, UK	Qualitative	Semi-structured interview	To explore CPs' awareness, and attitudes of intermediate care services	CPs	16	**Awareness:** CPs have limited involvement with intermediate care (IC) services<br>**Attitudes:** There is a need for improvement of effective communication of patients' medication information between secondary care, IC and community pharmacy
Laurence Guillaumie,2015	Canada	Qualitative	Focus groups interviews	To describe pharmacists' perceptions of patients having antidepressant therapy	CPs	43	**Attitudes:** Pharmacists showed a positive attitude toward their role in relation to patients with antidepressant therapy (ADT) and they appeared to be committed, especially at treatment initiation and monitoring side effects<br>**Perceptions:** However, pharmacists appeared to lack confidence and appropriate tools to carry out drug therapy monitoring, and especially to intervene systematically in adherence and monitor effectiveness

June Tordoff,2012	New Zealand	Mixed methods	Questionnaire Interviews	To explore perceptions of CPs of services that benefit older people	CPs	CS 403+QU 20	**Survey findings:** all pharmacies provide some baseline services (advice, dispensing of prescriptions, medicines disposal)<br>Adherence to medicines was supported by compliance packaging (96%), medication review (Medicines Use Review, MUR) (28%), and repeat prescription reminders (27%)<br>Thirty-five percent provided screening(e.g. cholesterol, blood pressure), and 32% provided medicines education to community groups<br>**Perceptions:** Compliance packaging and home delivery were thought the services most beneficial for older people, and should help people adhere to their medicines<br>**Interviews findings:** services provided are MUR, services to residential homes, visiting educators/special clinics, INR monitoring, services to hospices, and flu vaccination.<br>**Perceptions:** Benefits perceived included improvements in adherence, patient safety, and patient-knowledge of medicines, and convenient access to services
Kebede Beyene,2020	New Zealand	Mixed methods	Questionnaire Interviews	To explore pharmacists' experience and attitudes of anticoagulation management services	CPs	CS 108+ QU 12	**Survey findings:**<br>**Attitudes:** CPs have high self-efficacy and motivation to provide community pharmacy anticoagulation management services (CPAMS) had increased job satisfaction<br>CPAMS improves the pharmacist-patient relationship, allowing them to assist patients with other aspects of their healthcare<br>The two most frequently reported barriers were remuneration and staffing of pharmacists<br>**Interviews findings:**<br>**Perceptions:** the number of enrolled patients, and staffing were the main factors perceived by CPs preventing the further uptake and implementation of CPAMS
Marguerite Sendall,2018	Australia	Mixed methods	Questionnaire Interviews	To evaluate CPs' perceptions, knowledge, and confidence to promote bowel cancer and breast cancer screening	CP	CS 27+ QU 5	**Survey findings:**<br>**Perceptions:** Most CPs (71%) either 'agreed' or 'strongly agreed' discussing health advice, such as cancer screening, with their consumers was valuable and integral to their broader role<br>60% described their confidence as 'average' or 'good' when discussing bowel and breast cancer screening and prevention with consumers<br>**Interviews findings:**<br>**Perceptions:** Most CPs described their confidence to promote bowel cancer and breast cancer screening as moderate, and consistently reflected they felt more knowledgeable and<br>confident about bowel cancer topics than breast cancer topics

CPs: community pharmacists, GPs: general practitioners, CS: cross-sectional, QU: qualitative, PC: pharmaceutical care.
