# Supplementary material for: Attitudes, awareness, and perceptions of general public and pharmacists toward the extended community pharmacy services and drive-thru pharmacy services: a systematic review
Source: J Pharm Policy Pract. 2023 Mar 2;16:37. doi: 10.1186/s40545-023-00525-4 (PMC9979876; doi:10.1186/s40545-023-00525-4)
Supplement: Supplementary file 2 — Additional file 2. Quality assessment details of included studies in this systematic review. [file 40545_2023_525_MOESM2_ESM.pdf]

Additional file 2: Quality assessment details of included studies in this systematic review.

Item	Number of studies achieved Yes	Number of studies achieved NR	Details of studies achieved NR: Author, Year
**Cross-Sectional studies**			
Was the population well defined?	49	0	
Was the study objective clearly specified?	49	0	
Were the participants selected in unbiased and random manner?	48	1	Marguerite Sendall,2018
Were inclusion and exclusion criteria for being in the study prespecified?	21	28	Anna Millar,2016, Rana Abu Farha,2017, Heather Barry,2013, Amibor Chiedu,2019, Carolina Ung,2016,Dorota Kopciuch,2021, Gholamhossein Mehralian,2015,Hasan AL-Behadily,2017,Ibrahim Rayes,2015,June Tordoff,2012, Ma'aji Usman,2014, Marguerite Sendall,2018, Mariam Dabbous,2019,Menghuan Song,2015, Menghuan Song,2017,Osama Ibrahim,2013, Ozlem Erdogan,2012, Rana Abu Farha,2019, Rania Ghanem,2020, Rohit Verma,2019, Rose Evans,2021, Salah AbuRuz,2012, Semira Beshir,2012, Semira Beshir,2014, Stefan Balkanski,2019, Tessa Hastings,2017, Yin Wong,2019, Zelal Kharaba,2020
Was the survey unbiased?	48	1	Ozlem Erdogan,2012
Was the sample size justification or power analysis carried out?	41	8	Amibor Chiedu,2019, June Tordoff,2012, Ma'aji Usman,2014, Nehad Ayoub,2016, Ozlem Erdogan,2012, Semira Beshir,2012, Semira Beshir,2014, Stefan Balkanski,2019
Was the survey pilot tested or reviewed prior to the use?	44	5	Marguerite Sendall,2018, Mariam Dabbous,2019, Ozlem Erdogan,2012, Stefan Balkanski,2019, Yasmeen Thandar,2019
Was appropriate statistical analysis used?	48	1	Menghuan Song,2015
Have ethical issues been taken into consideration?	49	0	
**Qualitative studies**			
Was there a clear statement of the aims of the research?	3	0	

Is a qualitative methodology appropriate?	3	0
Was the research design appropriate to address the aims of the research?	3	0
Was the recruitment strategy appropriate to the aims of the research?	3	0
Was the data collected in a way that addressed the research issue?	3	0
Has the relationship between researcher and participants been adequately considered?	3	0
Have ethical issues been taken into consideration?	3	0
Was the data analysis sufficiently rigorous?	3	0
Is there a clear statement of findings?	3	0
How valuable is the research?	3	0
**Mixed method studies**		
Is there an adequate rationale for using a mixed methods design to address the research question?	3	0
Are the different components of the study effectively integrated to answer the research question?	3	0
Are the outputs of the integration of qualitative and quantitative components adequately interpreted?	3	0
Are divergences and inconsistencies between	3	0

quantitative and qualitative results adequately addressed?		
Do the different components of the study adhere to the quality criteria of each tradition of the methods involved?	3	0

NR: not reported

Appendix 2. Quality assessment grading of included studies in this systematic review.

Author, Year	Study design	Quality Grade
Anna Millar,2016	CS	8
Seena A. Yousuf,2019	CS	9
Maguy El Hajj,2013	CS	9
Rana Abu Farha,2017	CS	8
Khawla Abu Hammour,2019	CS	9
Nur Akmar Taha,2016	CS	9
Anita Weidmann,2012	CS	9
Heather E. Barry,2013	CS	8
Nur Taha,2014	CS	9
Abdul Nazer Ali,2017	CS	9
Ali Blebil,2020	CS	9
Aline Hajj,2019	CS	9
Amibor Chiedu,2019	CS	7
Amutha Selvaraj,2019	CS	9
Carolina Oi Lam Ung,2016	CS	8
Dorota Kopciuch,2021	CS	8
Gholamhossein Mehralian,2015	CS	8
Hasan H. AL-Behadily,2017	CS	8
Hee Peng Sia,2020	CS	9
Ibrahim Rayes,2015	CS	8
Jezreel Francis,2021	CS	9
Kofi B Mensah,2020	CS	9
M Zakour Khadari,2021	CS	9
M. Medhat,2020	CS	9
MA’AJI Usman,2014	CS	7
Mariam K Dabbous,2019	CS	8
Menghuan Song,2015	CS	8
Menghuan Song,2017	CS	9

Nehad M. Ayoub,2016	CS	8
Ogochukwu Offu,2015	CS	9
Osama Ibrahim,2013	CS	9
Ozlem Erdogan,2012	CS	6
Ramzi Shawahna,2021	CS	9
Rana Abu Farha,2019	CS	9
Rania E. Ghanem,2020	CS	9
Rohit Kumar Verma,2019	CS	9
Rose Evans,2021	CS	9
Salah AbuRuz,2012	CS	9
Samir Sakka,2022	CS	9
Semira A Beshir,2012	CS	8
Semira A. Beshir,2014	CS	8
Stefan Balkanski,2019	CS	7
Sujyoti Shakya,2020	CS	9
Tareq L. Mukattash,2018	CS	9
Tessa J. Hastings,2017	CS	9
Yasmeen Thandar,2019	CS	8
Yin Wong,2019	CS	9
Zelal Kharaba,2020	CS	9
Alamin Alabid,2021	CS	9
Furqan K. Hashmi,2017	Qualitative	10
Anna Millar,2016	Qualitative	10
Laurence Guillaumie,2015	Qualitative	10
June Tordoff,2012	Mixed methods	5
Kebede Beyene,2020	Mixed methods	5
Marguerite Sendall,2018	Mixed methods	5
