# Supplementary material for: Attitudes, awareness, and perceptions of general public and pharmacists toward the extended community pharmacy services and drive-thru pharmacy services: a systematic review
Source: J Pharm Policy Pract. 2023 Mar 2;16:37. doi: 10.1186/s40545-023-00525-4 (PMC9979876; doi:10.1186/s40545-023-00525-4)
Supplement: Supplementary file 3 — Additional file 3. Quality assessment grading of included studies in this systematic review. [file 40545_2023_525_MOESM3_ESM.pdf]

Additional file 3. Quality assessment grading of included studies in this systematic review.

Author, Year	Study design	Grade by reviewer #1	Grade by reviewer #2
Anna Millar,2016	CS	8	8
Seena A. Yousuf,2019	CS	9	9
Maguy El Hajj,2013	CS	9	9
Rana Abu Farha,2017	CS	8	8
Khawla Abu Hammour,2019	CS	9	9
Nur Akmar Taha,2016	CS	9	9
Anita Weidmann,2012	CS	9	9
Heather E. Barry,2013	CS	8	8
Nur Taha,2014	CS	9	9
Abdul Nazer Ali,2017	CS	9	9
Ali Blebil,2020	CS	9	9
Aline Hajj,2019	CS	9	9
Amibor Chiedu,2019	CS	7	7
Amutha Selvaraj,2019	CS	9	9
Carolina Oi Lam Ung,2016	CS	8	8
Dorota Kopciuch,2021	CS	8	8
Gholamhossein Mehralian,2015	CS	8	8
Hasan H. AL-Behadily,2017	CS	8	8
Hee Peng Sia,2020	CS	9	9
Ibrahim Rayes,2015	CS	8	8
Jezreel Francis,2021	CS	9	9
Kofi B Mensah,2020	CS	9	9
M Zakour Khadari,2021	CS	9	9
M. Medhat,2020	CS	9	9
MA’AJI Usman,2014	CS	7	7
Mariam K Dabbous,2019	CS	8	8
Menghuan Song,2015	CS	8	8
Menghuan Song,2017	CS	9	9

Nehad M. Ayoub,2016	CS	8	8
Ogochukwu Offu,2015	CS	9	9
Osama Ibrahim,2013	CS	9	9
Ozlem Erdogan,2012	CS	6	6
Ramzi Shawahna,2021	CS	9	9
Rana Abu Farha,2019	CS	9	9
Rania E. Ghanem,2020	CS	9	9
Rohit Kumar Verma,2019	CS	9	9
Rose Evans,2021	CS	9	9
Salah AbuRuz,2012	CS	9	9
Samir Sakka,2022	CS	9	9
Semira A Beshir,2012	CS	8	8
Semira A. Beshir,2014	CS	8	8
Stefan Balkanski,2019	CS	7	7
Sujyoti Shakya,2020	CS	9	9
Tareq L. Mukattash,2018	CS	9	9
Tessa J. Hastings,2017	CS	9	9
Yasmeen Thandar,2019	CS	8	8
Yin Wong,2019	CS	9	9
Zelal Kharaba,2020	CS	9	9
Alamin Alabid,2021	CS	9	9
Furqan K. Hashmi,2017	Qualitative	10	10
Anna Millar,2016	Qualitative	10	10
Laurence Guillaumie,2015	Qualitative	10	10
June Tordoff,2012	Mixed methods	5	5
Kebede Beyene,2020	Mixed methods	5	5
Marguerite Sendall,2018	Mixed methods	5	5

CS: Cross-sectional, Grading score for CS out of 9, Grading score for qualitative out of 10, Grading score for mixed-methods out of 5.
